# Supplementary material for: Maternal and Neonatal Outcome after the Use of G-CSF for Cancer Treatment during Pregnancy
Source: Cancers (Basel). 2021 Mar 10;13(6):1214. doi: 10.3390/cancers13061214 (PMC8001066; doi:10.3390/cancers13061214)
Supplement: Supplementary file 1 [file cancers-13-01214-s001.pdf]

cancers

MDPI

Article

Supplemental Materials: Maternal and Neonatal Outcome after the Use of G-CSF for Cancer Treatment during Pregnancy

Claudia Berends, Charlotte Maggen, Christianne A. R. Lok, Mathilde van Gerwen, Ingrid A. Boere, Vera E. R. A. Wolters, Kristel Van Calsteren, Heidi Segers, Marry van den Heuvel-Eibrink, Rebecca C. Painter, Mina Mhallem Gziri and Frédéric Amant

Supplementary Table S1. Pediatric follow-up of children prenatally exposed to G-CSF (*n* = 21).

Case	GA at Diagnosis (weeks + days)	GA at Delivery (weeks + days)	GA at Start Treatment (weeks + days)	Chemotherapy during Pregnancy (# of cycles)	G-CSF (Administrations)	Diagnosis	Age at Follow-Up	General Outcome	Cardiac Outcome	Neurological Outcome	Cognitive Outcome
1	15+5	38+2	21+6	EC q3w *(4)* Paclitaxel q1w (7)	Peg (4)	Breast Cancer	18 months	Normal, delay in motor development	*	0	0
							3 years	0	0	0	0
2	30+3	37+1	33+4	EC q3w(1)	Peg (1)	Breast Cancer	18 months	*	*	*	Delay in cognitive development
							3 years	*	0	*	0
							6 years	0	0	0	0
3	21+4	37+3	21+5	RC q3w (6)	Peg (2)	Non hodgkin lymphoma	18 months	Normal, delay in motor development	*	0	0
							3 years	0	0	0	0
4	25+1	34+2	25+3	AC q3w (3)	Peg (?)	Breast Cancer	18 months	0	0	0	0
							6 years	0	0	0	0
							9 years	0	0	0	0
5	*7+0*	*35+4*	*17+1*	FAC q3w (6)	Peg (2)	Breast Cancer	18 months	*	0	*	0
							6 years	*	0	*	0
							9 years	0	*	0	0
6	7+5	35+4	16+1	EC q2w (4) Pac q1w (1) Doc q3w (3)	Peg (4)	Breast Cancer	18 months	0	*	0	0
							3 years	Overweight, normal development	0	0	Delay in cognitive and language development
7	15+4	38+3	20+4	Doc q2w (3)	Peg (5)	Breast Cancer	18 months	0	*	*	Delay in cognitive development
							3 years	0	0	0	0
							6 years	0	0	0	0
8	18+0	36+1	18+5	R-ACVBP q3w (4) R-IE q3w (2)	Fil (?)	Non hodgkin lymphoma	18 months	0	*	0	0
9	6+2	36+6	12+6	AC q2w (4) Pac q1w (12)	Peg(4)	Breast Cancer	3 months	0	0	0	*
10	30+2	36+5	31+2	AC q2w (3)	Peg (3)	Breast Cancer	2 months	0	0	0	*
11	29+6	38+5	31+5	AC q2w (3)	Peg (3)	Breast Cancer	6 months	0	0	0	*
							14 months	0	*	0	0
12	28+1	36+0	*28+5*	AC q2w (3)	Peg (3)	Breast Cancer	18 months	0	0	0	Normal cognitive development for his premature age
13		38+2	35+0	AC q2w (2)	Lip (2)	Breast Cancer	3 years	0	0	0	0

14	22+3	37+2	39+6	AC q2w (4)	Peg (4)	Breast Cancer	3 years	0	0	0	0
15	17+6	37+4	22+1	AC q2w (4)<br>Pac q1w (7)	Lip (4)	Breast Cancer	11 months	Normal, delay in motor development	0	0	*
							18 months	0	*	0	0
16	17+5	36+5	18+1	AC q2w (4)<br>Pac (9)	Lip (4)	Breast Cancer	12 months	0	0	*	*
							18 months	0	*	0	*
17	16+6	37+0	22+1	AC q2w (4)<br>Pac (6)	Lip (4)	Breast Cancer	18 months	0	0	0	0
18	16+1	37+1	18+3	AC q2w (4)<br>Pac (9)	Lip (4)	Breast Cancer	18 months	0	*	0	0
19	16+5	38+3	18+1	R-CHOP q2w (6)	Peg (?)	Non hodgkin lymphoma	3 years	*	*	*	0
							6 years	0	0	0	0
20	15+2	29+2	16+0	R-CHOP q2w (6)<br>VIM q1w (1)	Peg (?)	Non hodgkin lymphoma	18 months	0	0	0	Normal cognitive development, delay in language development
21	14+6	38+2	16+1	AC q2w (4)<br>Pac q1w (8)	Lip (4)	Breast Cancer	18 months	Normal, delay in motor development	*	0	0
							3 years	0	0	0	0

0: Normal development (in pediatric consultation), no abnormalities in cardiac outcome and neurological outcome, normal cognitive development
*: Data not available
All breast cancers are invasive ductal adenocarcinoma
Abbreviations chemotherapy: AC: doxorubicin-cyclophosphamide; doc: docetaxel; EC: epirubicin-cyclophosphamide; FAC: fluorouracil-doxorubicin-cyclophosphamide; pac: paclitaxel; R-CHOP: rituximab+cyclofosfamide+doxorubicine+vincristine + prednisone; R-ACVBP: rituximab, doxorubicin, cyclophosphamide, vindesine, bleomycin + prednisone; R-IE: Rituximab, Ifosfamide, Etoposide; VIM: ifosfamide, mitoxantrone;
General abbreviatons : GA: gestational age; q1w: every week; q2w: every 2 weeks; q3w: every 3 weeks; ?: unknown number of cycles/administrations; G-CSF: fil: filgrastim; lip: lipefilgrastim; peg: pegfilgrastim
OVERVIEW of examinations in INCIP follow-up study
Pediatric consultation: A general physical examination and neurological assessment performed by a pediatrician.
Cardiac assessment: 12-lead electrocardiograph (ECG) and a full echocardiographic assessment for structural and functional characteristics was collected by a cardiologist/experienced sonographer.
Cognitive assessment: an age-adapted test battery for the assessment of intelligence, verbal and non-verbal memory, attention, working memory and executive functions by an experienced psychologist (Bayley Scales of Infant and Toddler Development, third edition (BSID-III), Child Behavior Checklist (CBCL), Behavior Rating Inventory of Executive Function – Preschool Version (BRIEF-P), Wechsler Preschool and Primary Scale of Intelligence, third edition (WPPSI-III), Subtask of Children's Memory Scale (CMS), Subtasks of Amsterdam Neuropsychological Tasks (ANT), Behavior Rating Inventory of Executive Function (BRIEF))
